# Different mechanisms of serum complement activation in the plasma of common (*Chelydra serpentina*) and alligator (*Macrochelys temminckii*) snapping turtles

**DOI:** 10.1371/journal.pone.0217626

**Published:** 2019-06-06

**Authors:** Sarah Baker, Ethan Kessler, Lancia Darville-Bowleg, Mark Merchant

**Affiliations:** 1 Illinois Natural History Survey, Champaign, Illinois, United States of America; 2 Moffitt Cancer Center and Research Institute, Tampa, Florida, United States of America; 3 Department of Chemistry, McNeese State University, Lake Charles, Louisiana, United States of America; Chang Gung University, TAIWAN

## Abstract

Reptiles are declining worldwide yet our understanding of their immune function lags far behind other taxa. The innate immune system is the primary mode of defense in reptiles, and the serum complement cascade is its major component. We assessed serum complement activity of plasma in two closely related aquatic turtle species, the common snapping turtle (CST; *Chelydra serpentina*) and alligator snapping turtle (AST; *Macrochelys temminckii*). We used a sheep red blood cell (SRBC) hemolysis assay to assess serum complement activity. Although the antibacterial activities of the plasma of these turtle species are similar, the hemolytic activity was much stronger in CST than AST. Treatment with inhibitors of the serum complement cascade indicated differences in the mechanisms of complement activation between the turtle species. We subjected plasma from both turtle species to mannan affinity chromatography and analyzed the eluate with SDS-PAGE, which revealed that plasma from the CSTs contained only small amounts of one C-type lectin protein while the AST plasma contained high concentrations of two C-type lectins (31.0 and 35.9 kDa). Edman degradation analyses confirmed that the two AST proteins contained identical N-terminal sequences. Thus, the CST appears to rely more heavily on the alternative mechanism of serum complement activation, while the AST appears to rely more on the lectin-mediated pathway, which is a pattern recognition response to prokaryotes not activated by the SRBCs. These results are unique in that the use of serum complement pathways are generally assumed to be conserved within clades.

## Introduction

The animal immune system is divided into two branches, innate and adaptive immunity, which work both individually and in tandem to provide defense from pathogens. Innate immunity is nonspecific, requires no previous exposure, and responds rapidly. In contrast, adaptive immunity requires previous exposure, is very specific, and may take 48 hours or more to launch a coordinated response. The primary mechanism of the innate immune response is the serum complement system. Activation of the serum complement cascade leads to eventual construction of a multiprotein membrane attack complex in the membrane of microbes that causes leakage of cellular contents, and eventual lysis [1]. In addition, serum complement exhibits a wide array of other functions, such as opsonization, phagocytosis, chemotactic recruitment of leukocytes, inflammation, and expression of proinflammatory cytokines, which facilitate a multifaceted immune response [2]. Serum complement components are also crucial for coordinating some important events in adaptive immune response [3].

The serum complement cascade is activated by three different mechanisms, the classical, alternative, and lectin pathways. The classical pathway involves the recognition and binding of antigen by antibodies, and thus acts as a bridge between innate and adaptive immunity [4]. The alternative pathway involves activation via autolytic cleavage of an internal thioester in the complement C3 protein [5–6]. The lectin pathway is activated when a C-type lectin recognizes and binds to a carbohydrate moiety on the surface of a microbe [7–8]. All three modes of activation converge to a common mechanism and culminate in the formation of a multiprotein complex on the outer membrane of microbes which causes lysis and leakage of cellular contents [9].

Ectothermic vertebrates are typically thought to have a more effective innate immunity and less advanced adaptive immune responses [10–11]. Compared to other taxa, few studies have been conducted quantifying innate immunity in reptiles, and filling this knowledge gap has been identified as a priority research area [12]. Reptile population declines have been documented worldwide with factors such as disease and environmental pollution identified as major threats [13]. Turtles are arguably the most imperiled group of vertebrates in the world, with approximately 61% of species threatened or extinct [14]. Therefore, understanding reptile immune system function may be important for management of current and emerging threats. Serum complement has been studied extensively in crocodylians [15–19], with fewer studies directly evaluating it in turtles [20–21].

Chelydridae is a family of turtles of two genera; *Chelydra* (Common Snapping Turtles; CST) and *Macrochelys* (Alligator Snapping Turtles; AST). Chelydridae split from other turtle families roughly 110 MYA and the last common ancestor of CSTs and ASTs likely existed 21 MYA [22]. These two genera share many characteristics and are broadly referred to as snapping turtles. While they exist in sympatry throughout the southeastern United States, CSTs are true aquatic generalists whereas ASTs are habitat specialists, preferring flowing waters and oxbows with abundant structure [23]. Additionally, the CST ranges from Canada to northern South America whereas the AST is found only in the Gulf of Mexico drainage in southeastern United States [24]. Previous work has shown that CSTs have a relatively more robust innate immune response when compared to ASTs, likely due to differences in geographic range and habitat requirements [25]. Additionally, CSTs are abundant throughout much of their range, while ASTs are under review for listing under the US endangered species act and classified as vulnerable by the IUCN Tortoise and Freshwater Turtle Specialist Group [26]. This study evaluated serum complement immune activity of snapping turtles (Chelydridae) in response to time and temperature, and investigated the primary activation pathway used by each species.

## Materials and methods

*Chemicals and Biochemicals*–Ethylene diamine tetraacetate (EDTA), magnesium chloride, calcium chloride, methylamine, salicylaldoxime, Coomassie blue protein stain, mannose-agarose, and a protease isolated from *Streptomyces griseus* (P8811) were purchased from Sigma-Aldrich (St. Louis, MO, USA). Sheep red blood cells (10%, v/v) were purchased from Rockland Immunochemicals (Rockland, MD, USA).

*Treatment of animals*–All work was conducted under approved University of Illinois IACUC protocol #18000. Wild turtles were collected under Illinois Department of Natural Resources permits #NH18.6224 and #15–008. We captured alligator snapping turtles in Union County, Illinois. We trapped common snapping turtles using double-throated hoop nets (0.6 m diameter) in Blackwell Forest Preserve (41.84021, -88.179047) and Pratt’s Wayne Woods (41.958355, -88.229100) in DuPage County, Illinois, and Crystal Lake Park (40.122892, -88.209027) in Champaign County, Illinois. We collected blood samples from each animal from the dorsal caudal vein using a 22 ga. needle and 3 mL syringe. We pooled plasma from all individuals of each species to minimize individual variation, divided pooled plasma into 5 mL aliquots, and stored plasma at -20°C until we conducted the assays. As date of capture for individual turtles varied, time frozen also varied, but did not exceed 8 weeks for any individual sample which should not significantly decrease activity [27]. Immediately prior to each assay, plasma was placed in a 30°C water bath to minimize the time required to thaw.

*Sheep red blood cell (SRBC) hemolysis assay*–We exposed pooled plasma samples from ASTs and CSTs to 1% SRBCs (v/v) to determine hemolytic capacity of plasma components. Volume-dependent assays were conducted by incubating different volumes of plasma, diluted with unbuffered saline, with SRBCs at ambient temperature (25°C) for 30 min. The samples were centrifuged at 2500 x*g* and 150 μL of the resulting supernatants were removed to a 96-well plate. We determined the optical density of each sample at 540 nm using a Benchmark Plus (BioRad, Hercules, CA, USA) microtiter plate reader.

We determined the kinetic characteristics of SRBC hemolysis for plasma from each turtle species by incubation of 3.0 mL of plasma, 2.0 mL of unbuffered saline, and 5.0 mL of 1% SRBCs. Plasma dilution was chosen based on the results of the volume-dependent assays described above. Aliquots (250 μL) were removed at different time points (0, 2, 5, 10, 15, 20, 30, 60 min). We centrifuged the samples at 2500 x*g* and removed the supernatants to a 96-well plate as described above.

To determine the effects of temperature on the hemolytic activity, we acclimated plasma samples diluted with unbuffered saline at various temperatures (5–40°C) for 10 minutes. We then exposed the plasma to SRBCs for 30 min, centrifuged at 2500 x*g*, and removed the supernatants to a 96-well plate and the OD_540_ measured as above.

*Mannan affinity chromatography*–We equilibrated a mannose-agarose suspension (3 mL) with 3 mL of mixing buffer (10 mM Tris–HCl, pH 7.8, 1.25M NaCl) and allowed it to settle into a column prepared from a Pasteur pipette plugged with cotton. Five mL of AST or CST plasma were mixed with 5 mL of loading buffer (20 mM Tris–HCl, pH 7.8, 2.5M NaCl). The diluted plasma was allowed to filter through the column and the column was washed with 10 volumes of loading buffer. We eluted the proteins using 5 mL of elution buffer (10 mM Tris–HCl, pH 7.8, 1.25M NaCl, 2 mM EDTA), and the resulting eluates were transferred to 1.0 kDa centrifugal concentrator tubes (Centriprep, Millipore Corp., Billerica, MA, USA) and centrifuged at 7500 x*g* to concentrate. When the samples reached minimal volume, they were desalted by the addition of 10 mL of distilled water and centrifuged at 7500 x*g* for 2 hours.

*SDS-PAGE analysis*–The desalted eluates (20 μL) were mixed with 20 μL of SDS sample loading buffer (BioRad, Hercules, CA, USA), incubated at 95°C for 5 min., cooled on ice for 5 min, then centrifuged at 16,000 x*g* for 30 seconds. We loaded samples (35 μL) and PageRuler Plus prestained protein markers (Fisher Scientific, Chicago, IL, USA) onto 4–20% polyacrylamide precast gradient gels (BioRad, Hercules, CA, USA) and resolved at 20 V/cm for 90 min. The gels were stained for 10 min with Coomassie blue R-250 and destained in a solution of 45% H_2_0, 45% MeOH, and 10% glacial AcOH (v/v) for three hours.

*N-terminal sequencing*–The affinity-purified proteins resolved in the SDS polyacrylamide gel were electroblotted to a PVDF membrane and the amino acid sequence determined by Edman degradation on a Procise 494HT protein sequencing system (ThermoFisher Scientific, Waltham, MA, USA).

*MALDI TOF analysis*—The protein samples were desalted, concentrated and spotted on a ground-steel plate using a 1:1 ratio (v/v) of sample and saturated sinapinic acid (SA) matrix. We determined molecular masses using linear mode matrix-assisted laser desorption ionization time-of-flight (MALDI-TOF) mass spectrometry (MALDI-TOF/TOF—UltrafleXtreme, Bruker Daltonics, Billerica, MA, USA). The samples were analyzed in positive ion mode and 500 shots collected. To collect mass spectra we used a mass range from 20 to 200 kDa. Spectra were acquired and the exact protein masses were determined using FlexAnalysis software (Version 2.0, Bruker Daltonics).

*Statistics and controls*–For each SRBC hemolysis assay, we obtained a complete lysis positive control by rapidly syringing a suspension of 1% (v/v) SRBCs in water rapidly through a 31 ga needle. This action resulted in 100% hemolysis, as confirmed by inspection under 400x magnification under a phase contrast microscope. The control sample was used as a 100% lysis control for comparison to all other samples. In addition, every SRBC hemolysis assay included four replicates with no plasma added. The results, which account for the spontaneous lysis of SRBCs, were subtracted from the result for each independent treatment, thereby removing the influence of autohemolysis. Samples for each assay were analyzed in quadruplicate so that valid statistical analyses could be conducted. All results presented represent the means ± standard deviations for four independent determination.

## Results

**Fig 1:**
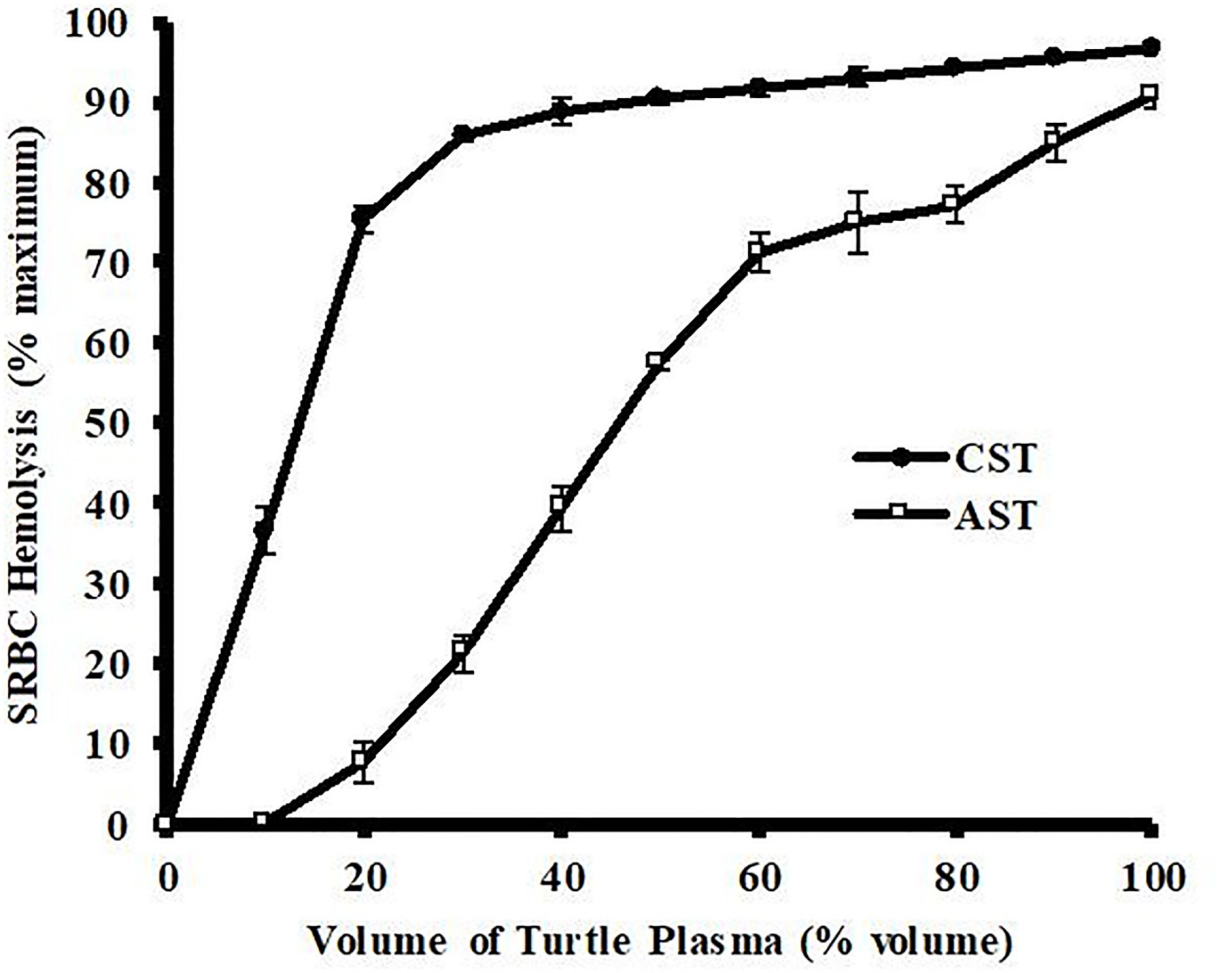
Volume-dependent hemolysis of SRBCS by plasma from Common (CST) and Alligator (AST) Snapping Turtles. Different volumes of pooled plasma samples derived from ASTs and CSTs diluted in buffer were incubated with 1% SRBCs for 30 min at ambient temperature. Hemolysis activity was determined by spectrophotometry at 540 nm and expressed as % maximum as compared to a complete hemolysis positive control. The data represent the means ± standard deviations for four independent determinations.

The plasma from ASTs and CSTs both exhibited volume-dependent hemolysis of 1% SRBCs (Fig 1). Plasma from CSTs showed much higher activity than that from ASTs. The plasma from CSTs showed a biphasic, asymptotic-like response with linear hemolysis between 0 and 20 μL (slope = +3.75% hemolysis/μL plasma) and gradually declining increases thereafter. In comparison, the plasma from ASTs showed a near linear response from 10–60 mL, but with a much lower slope of +1.2% hemolysis/μL plasma. The hemolytic activity of the 100% AST plasma (90.7 ± 1.5%) was similar to that of the 50% CST plasma (90.6 ± 0.9%).

**Fig 2:**
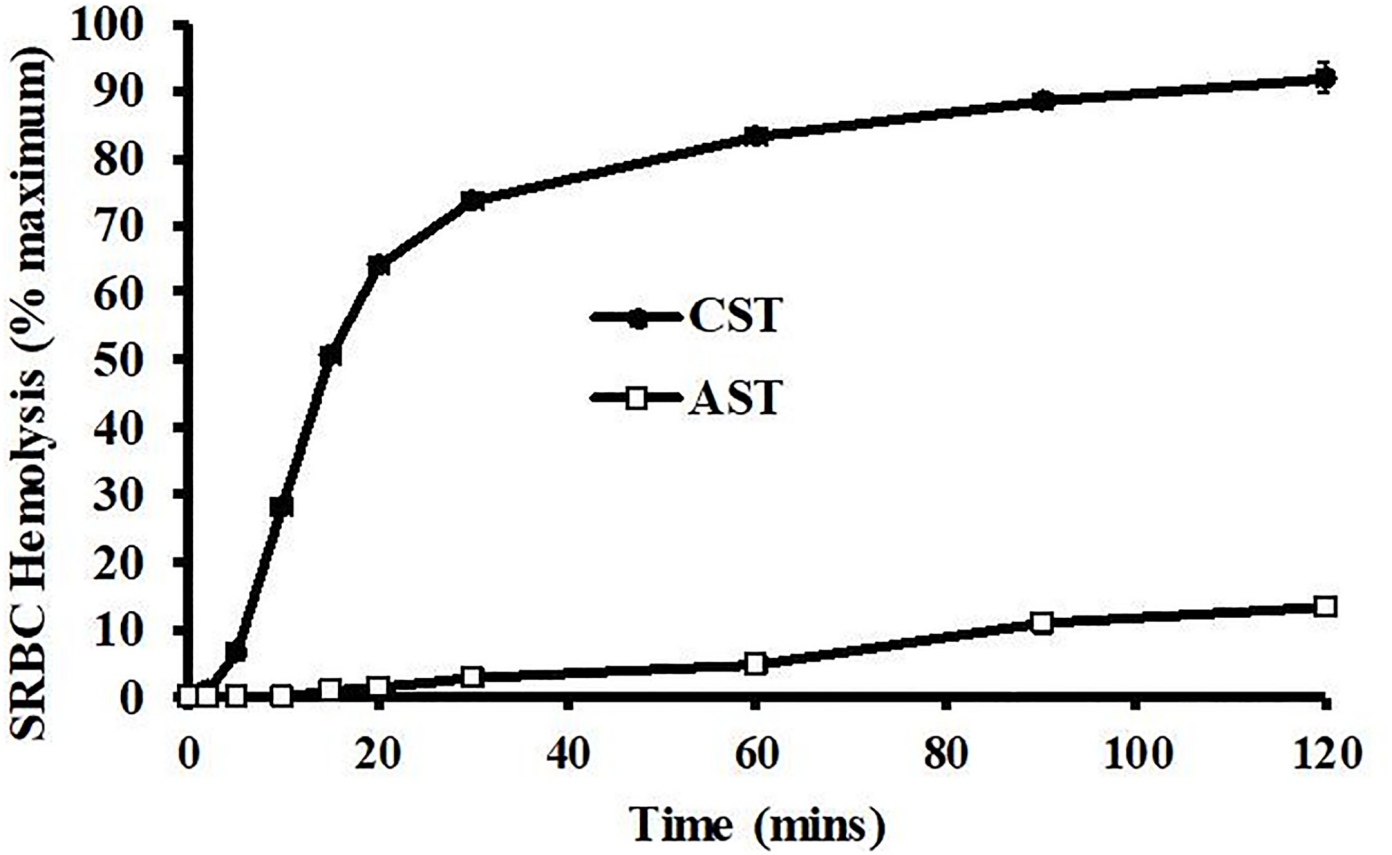
Kinetic analysis of SRBC hemolysis by plasma from Common (CST) and Alligator (AST) Snapping Turtles. Pooled plasma samples derived from ASTs and CSTs (30% v/v) diluted in unbuffered saline were incubated with 1% SRBCs for different amounts of time. Hemolysis activity was determined by spectrophotometry at 540 nm and expressed as % maximum as compared to a complete hemolysis positive control. The data represent the means ± standard deviations for four independent determinations.

At an assay volume of 30%, the CST and AST plasma samples showed drastic differences in the kinetic properties of hemolytic activity (Fig 2). The CST plasma showed 50-fold higher hemolytic activity than that of AST plasma at 15 and 20 min. The CST plasma exhibited near maximal activity at 30–60 min., but the plasma from ASTs only showed 13.2 ± 0.3% activity at 120 min. The initial hemolytic activity (2–20 min) of the CST show an approximate 50-fold increase in slop compared that of plasma from ASTs.

**Fig 3:**
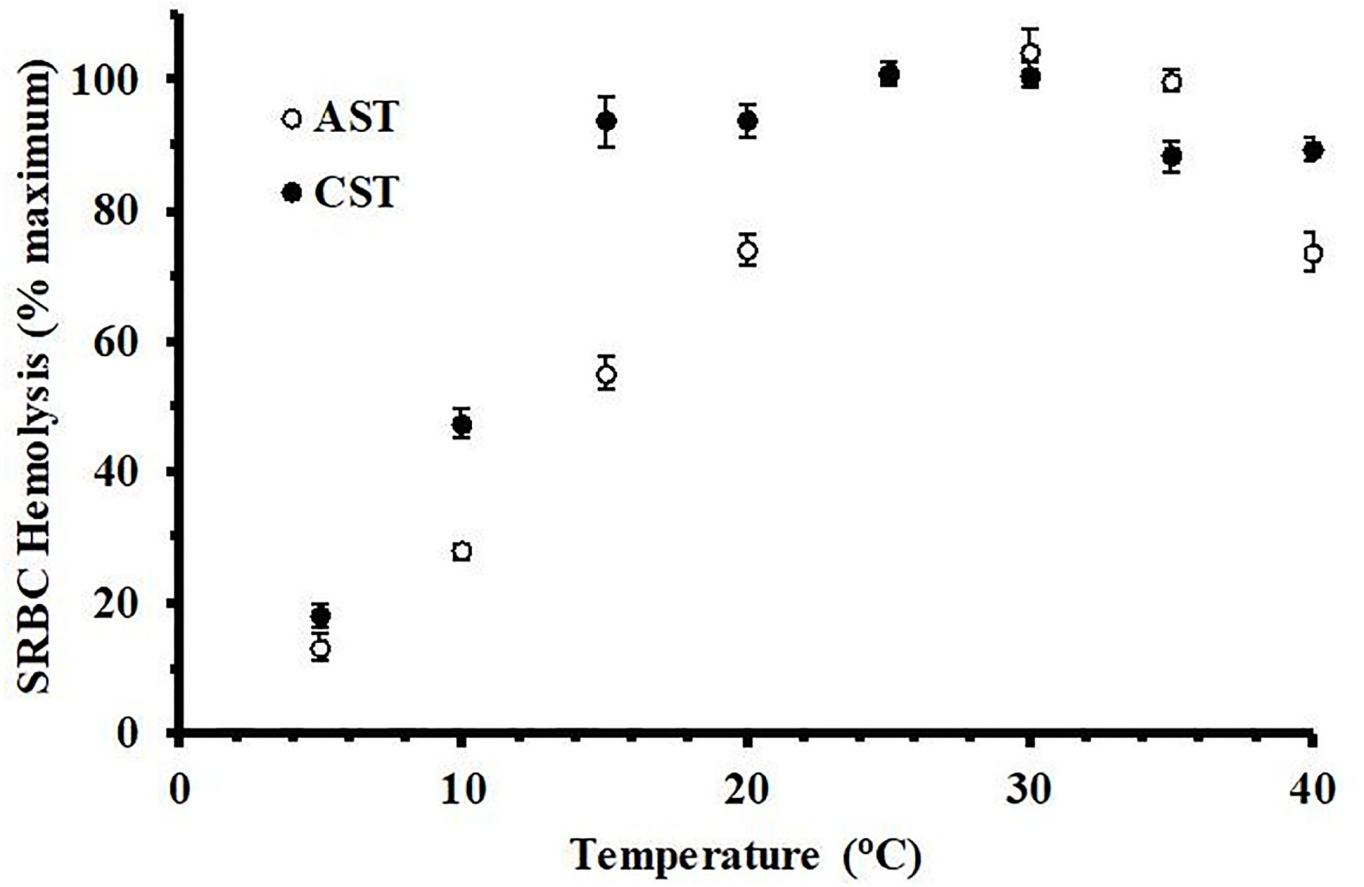
Temperature-dependent hemolysis of SRBCs by plasma from Common and Alligator Snapping Turtles. Pooled plasma samples derived from ASTs (60%, v/v) and CSTs (20%, v/v) diluted in unbuffered saline were incubated with 1% SRBCs at different temperatures. Hemolysis activity was determined by spectrophotometry at 540 nm and expressed as % maximum as compared to a complete hemolysis positive control. The data represent the means ± standard deviations for four independent determinations. *indicates statistically lower (p ≤ 0.05) than peak values for the respective species.

Incubation of AST and CST plasma with 1% SRBCs at various temperatures resulted in different thermal hemolysis profiles between the species (Fig 3). Plasma from CSTs showed a linear increase in hemolytic activity from 5–15°C, with a slope of 6.7% activity/°C, and remained at this peak level from 15–30°C. Plasma from ASTs achieved a similar peak activity, however the increase was more gradual, increasing in a linear fashion from 5–25°C with a lower slope of 3.9% hemolytic activity/°C and remaining at peak activity level from 25–35°C. Activity from CST plasma decreased slightly (p < 0.05) to 78.6 ± 2.3 and 81.2 ± 3.6% activity at 35 and 40°C, respectively. The activity of plasma from AST remained maximal at 35°C, but then declined sharply (p < 0.01) to 65.6 ± 2.6% at 40°C.

Hemolytic activity of plasma from both CST and AST is heat labile and sensitive to treatment with a generic protease. Mild heat treatment of plasma (56°C for 30 minutes) reduced SRBC hemolysis from 81.4 to 1.3% in AST and from 94.8 to 2.2% in CST (Table 1). Additionally, treatment of plasma with protease or 5mM EDTA eliminates nearly all hemolytic activity. Activity can be restored by the addition of Ca^2+^ or Mg^2+^, but not Ba^2+^, Cu^2+^, or Fe^2+^ (Table 2).

**Table 1:** Hemolysis inhibitors.

Treatment	CST SRBC Hemolysis (% max)	AST SRBC Hemolysis (%max)
Turtle plasma	94.8 ± 2.3	81.4 ± 0.8
Turtle plasma (56°C, 30 min)	2.2 ± 0.1	1.3 ± 0.1
Turtle plasma + 100 U protease	0.9 ± 0.1	0.4 ± 0
Plasma + 5 mM EDTA	4.1 ± 0.3	2.2 ± 0.3
Plasma + 20 mM phosphate	5.0 ± 0.3	2.7 ± 0.3
Plasma + 20 mM citrate	4.3 ± 0.1	2.7 ± 0.1
Plasma + 20 mM ammonia	93.1 ± 0.7	83.4 ± 1.5
Plasma + 20 mM methylamine	93.3 ± 1.1	83.0 ± 1.2
Plasma + 50 mM salicylaldoxime	9.8 ± 0.4	34.7 ± 0.5

Effects of mild heat treatment, protease, and chemical inhibitors on SRBC hemolysis by plasma from Common (*Chelydra serpentina*; CST) and Alligator (*Macrocheyls temminckii*; AST) snapping turtles

**Table 2:** Divalent metal ions.

Treatment	CST SRBC Hemolysis (% max)	AST SRBC Hemolysis (%max)
Turtle plasma	97.3 ± 2.7	84.4 ± 1.8
Plasma + 5 mM EDTA	3.2 ± 0.2	1.9 ± 0.2
Plasma + 5 mM EDTA + 10 mM MgCl_2_	96.8 ± 1.9	36.2 ± 1.0
Plasma + 5 mM EDTA + 10 mM CaCl_2_	98.8 ± 2.1	85.1 ± 0.9
Plasma + 5 mM EDTA + 10 mM BaCl_2_	2.9 ± 0.3	1.1 ± 0.2
Plasma + 5 mM EDTA + 10 mM CuCl_2_	2.2 ± 0.1	1.6 ± 0.3
Plasma + 5 mM EDTA + 10 mM FeCl_2_	3.7 ± 0.4	1.8 ± 0.2

Effects of specific divalent metal ions on complement-mediated SRBC hemolysis by plasma from Common (*Chelydra serpentina*; CST) and Alligator (*Macrocheyls temminckii*; AST) snapping turtles

**Fig 4:**
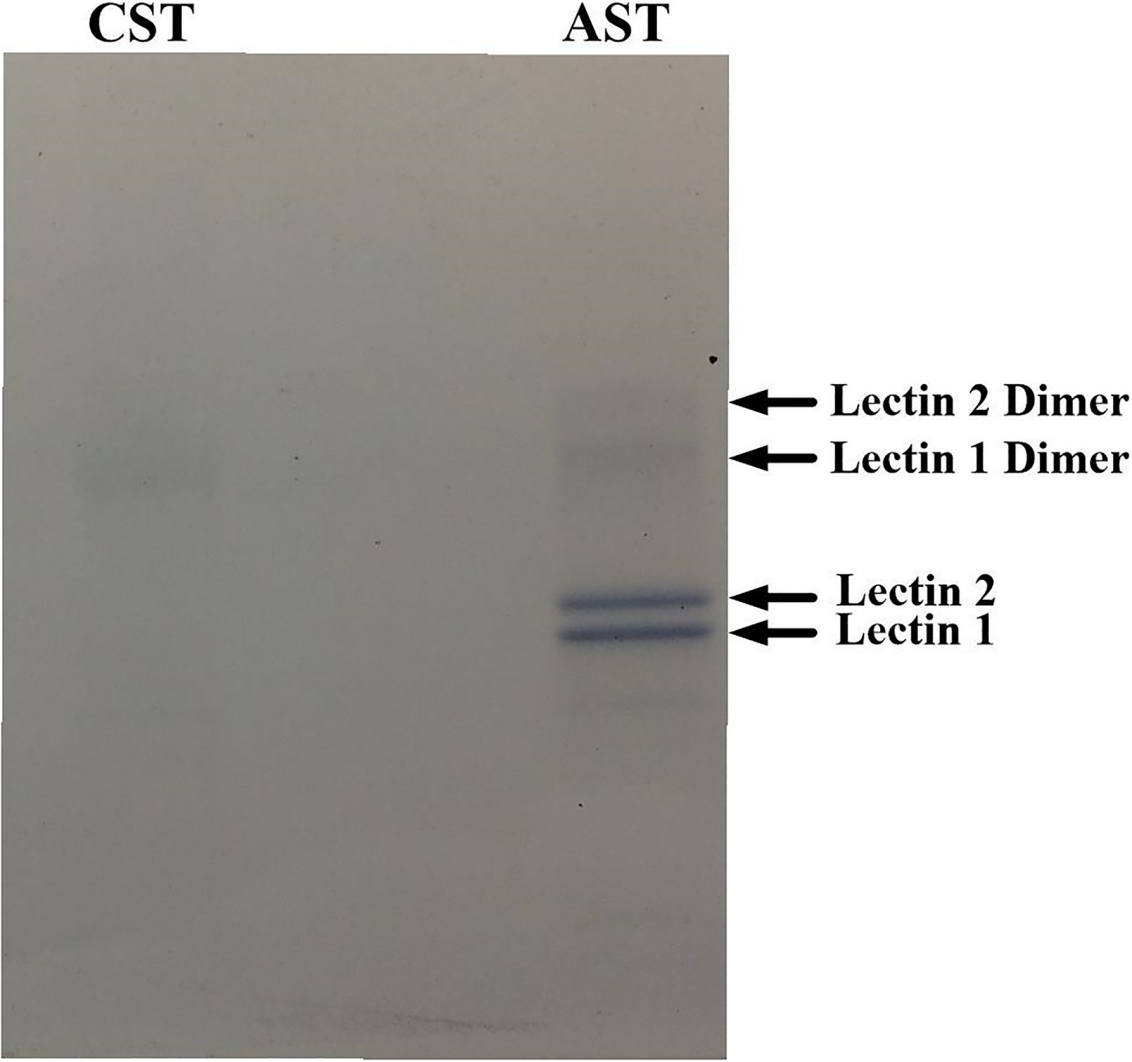
SDS-PAGE analysis of eluates from mannan-agarose affinity column. Plasma samples (5 mL) from Alligator and Common Snapping Turtles were filtered through a mannan-agarose affinity column in the presence of 20 mM Ca^2+^. Samples were eluted using EDTA buffer, concentrated and desalted using microcentrifugal concentrators, resolved by SDS PAGE, and stained using Coomassie blue.

**Fig 5:**
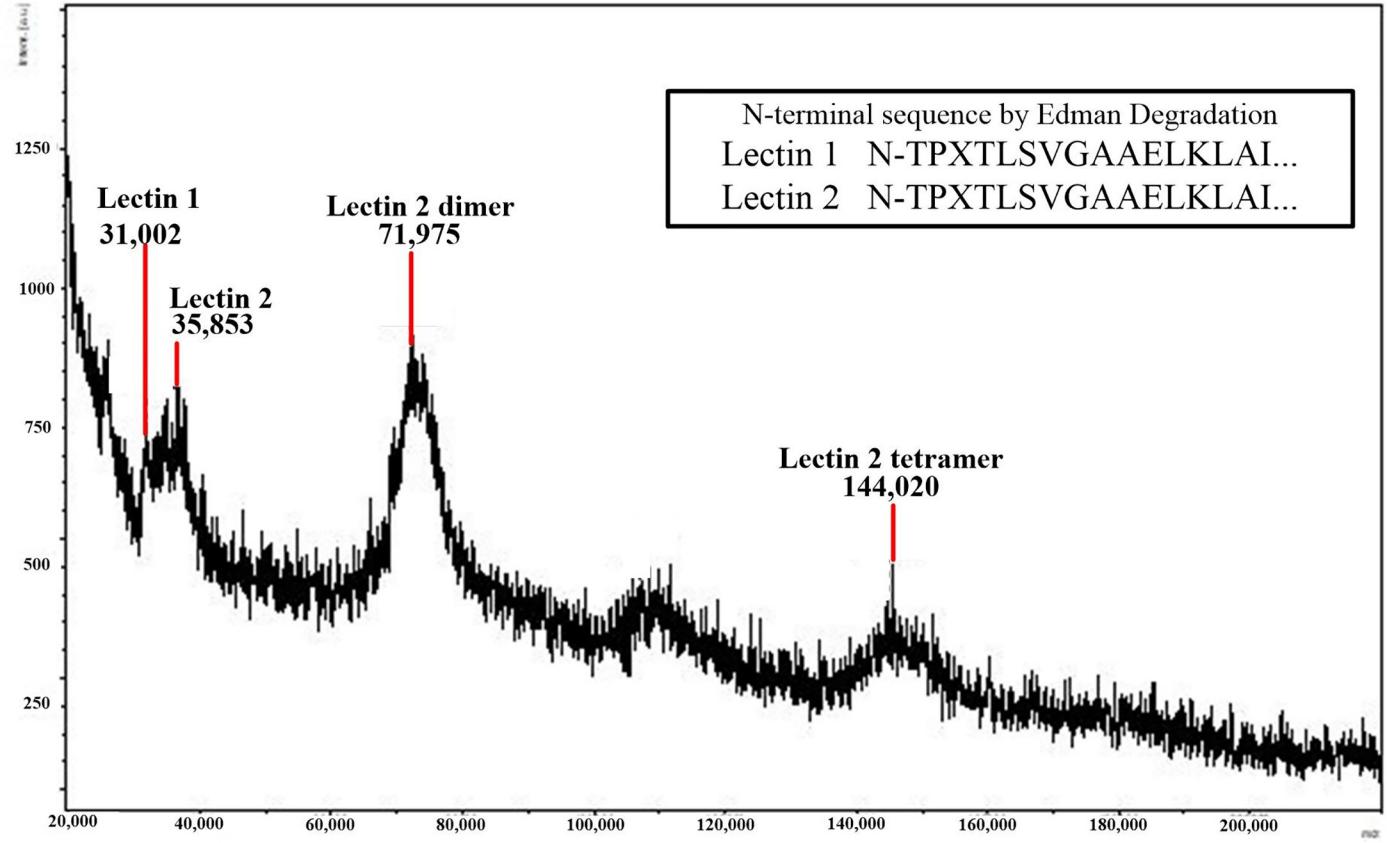
MALDI-TOF analysis of eluates from mannan-agarose affinity column. Plasma samples (5 mL) from Alligator Snapping Turtles were filtered through a mannan-agarose affinity column in the presence of 20 mM Ca^2+^. Samples were spotted with sinapinic acid and analyzed in the mass range of 20–200 kDa.

Mannan affinity chromatography and SDS page analysis indicated that the ASTs expressed high amounts of two proteins that interact with mannan (labeled as Lectin 1 and Lectin 2 in Figs 4 and 5), while the CST produced a low amount of one of the mannan-binding proteins. The MALDI-TOF analyses of these two proteins revealed Mr values of 31.0 and 35.9 kDa. The two proteins appeared to be present as dimers in the SDS-PAGE (Fig 4), but only the dimer and tetramer for Lectin 2 could be detected by MALDI (Fig 5). The Edman degradation analyses of the two mannan-binding proteins in AST plasma indicated that these proteins contain identical N-terminal sequences.

## Discussion

Incubation of plasma from CSTs or ASTs caused a volume-dependent hemolysis of SRBCs (Fig 1). The hemolytic activity was much more potent with the plasma of the CST, which was surprising because the results from a previous study found antibacterial activities of plasma from these turtle species was similar, with CSTs showing only slightly higher activity [25]. In addition, the kinetic analysis of the SRBC hemolysis by CST plasma was much faster and more potent than that from the AST (Fig 2). The activity of AST plasma remains low throughout the experiment, likely due to low concentrations of complement proteins preventing increased activity [28]. Although the hemolytic activities were very different, they are both mediated by the serum complement system of proteins because they were heat labile, sensitive to proteases, and inhibited by chelators of divalent metal ions (Table 1). These characteristics eliminate the possibility that the hemolysis was due to heat-stable antimicrobial cationic peptides [29], or lytic enzymes such as lysozyme that are not dependent on Mg^2+^ or Ca^2+^ for activity [30].

The thermal profiles of complement activity of these two turtle species reflect the differences in their latitudinal distributions. The CST can be found from the coast of the Gulf of Mexico to as far north as south-central Ontario, Canada [24]. The relatively high hemolytic activity of this species at 10–20°C is indicative of an ectotherm that must function in colder climates for a large portion of the year. For example, in a study at the northern end of the range for the CST, mean summer body temperatures were 22.7°C [31], which was far lower than their preferred body temperature of 28–30°C in a laboratory setting [32–33]. In contrast, the AST inhabits a more limited range, with little evidence of stable populations north of southeastern Missouri [23,34]. Although preferred body temperature data is unknown for this species, ASTs are exposed to warmer, more temperate conditions than northern populations of CSTs. Therefore, it is not surprising that the serum complement activity of this species is skewed toward higher temperatures and exhibits maximal activity at 35°C.

Since the plasma used in these assays came from animals which were not previously exposed to SRBCs, it is highly unlikely that it contained antibodies directed toward the SRBCs, precluding the possibility that the hemolytic activity exhibited by the plasma from either CSTs or ASTs was mediated by the classical pathway. Additionally, it is very unlikely that these animals would have had high titers of natural antibodies that would interact with SRBCs. The activity was also not affected by the presence of 20 mM methylamine (Table 1), a compound that is known to interfere with activity of complement protein C4 and inhibit the classical complement cascade [35–36] at relatively low concentrations. The sensitivity of the plasma to salicylaldoxime and EDTA strongly suggest that the activities are mediated by the alternative complement system. Salicylaldoxime is known to inhibit the alternative pathway [37], and EDTA sequesters Mg^2+^, which is known to be required for the interaction of C3b with factor B [38], an interaction critical for alternative complement function. However, the EDTA-inhibited hemolysis could be restored by the addition of either Mg^2+^ or Ca^2+^ in CST plasma, while the activity in AST plasma could only be restored by the addition of excess Ca^2+^. This indicates that hemolytic activity observed in the plasma from ASTs is not due to the classical or alternative mechanisms but is due to the lectin-mediated cascade.

Vertebrates produce lectins that recognize and bind mannan oligosaccharides that are expressed on the external surfaces of bacteria [39–40]. Once bound to oligosaccharide, the lectin-mediated complement pathway is activated. This is a component of the pattern recognition of the innate immune system whereby vertebrate proteins utilize specific molecular patterns to distinguish self from non-self tissues [41]. The results obtained from the mannan affinity column and SDS page indicated ASTs expressed high amounts of two proteins that interact with mannan in a Ca^2+^-dependent manner, while the CST produced a low amount of one of the mannan-binding proteins. This explains the observation that the hemolytic activity in AST plasma was inhibited by EDTA and restored by Ca^2+^, but not entirely by Mg^2+^ (Table 2), as the lectin pathway is mediated by a C-type lectin that requires Ca^2+^ for activity [42]. In addition, this also provides an explanation for the high antibacterial activity of AST plasma [25] while the SRBC hemolysis was low (Figs 1 and 2). Since the SRBCs are of eukaryotic origin, they lack mannan expression and thus do not elicit the lectin pathway of complement activation. Similar to other vertebrate systems, the AST proteins appear to dimerize and the dimers interact to form homotetramers (Fig 5) [43]. This is evident for Lectin 2; although the putative dimer for Lectin 1 can been seen on the SDS-PAGE analysis (Fig 4), the dimer and tetramer of Lectin 1 were not abundant enough to produce a signal using MALDI-TOF analysis (Fig 5), although the dimer for Lectin 2 can be visualized on the SDS-PAGE analysis (Fig 4). Although the Lectin 1 dimer was detected at the same approximate intensity as the monomer (Fig 4), the dimer was detected with a far lower intensity in the SDS-PAGE analysis (Fig 5). In addition, the Lectin 2 dimer was undetected in the MALDI analysis, although it was present in the same intensity as the Lectin 1 dimer in the SDS analysis. The differences in the sensitivity of these proteins to the MALDI analyses is unclear, but has been observed for other species (Darville et al., 2012).

Collectively, these data indicate that two closely related turtle species utilize different methods of complement activation. The primary complement defense mechanism for the CST is the alternative pathway, while the AST relies heavily on the lectin-mediated pathway. This would explain why the antibacterial activities for these two species were similar [25], but the SRBC hemolysis was much lower for the AST (Fig 1). Because SRBCs are of eukaryotic origin, they do not exhibit the mannan plasma membrane oligosaccharide pattern typical of prokaryotic organisms. Therefore, while the antibacterial activities of AST plasma were similar to CST plasma, the SRBC hemolysis values were far lower. In addition, the ASTs produced much more mannan-binding lectin protein than did CSTs (Fig 4, Table 2). It is unclear whether the difference in dominant complement pathway between CSTs and ASTs is due to genetic drift, habitat-driven phenotypes, or evolutionary adaptation to differential microbe exposure between the species. Future studies should focus on the investigation of complement activation cascades in other turtle species to determine if this phenomenon is widespread or confined to Chelydridae.

## Supporting information

S1 Fig Raw data used for calculation of volume-dependent hemolysis of SRBCS by plasma from Common and Alligator Snapping Turtles. (PDF)

S2 Fig Raw data used for calculation of kinetic analysis of SRBC hemolysis by plasma from Common and Alligator Snapping Turtles. (PDF)

S3 Fig Raw data used for calculation of temperature-dependent hemolysis of SRBCs by plasma from Common and Alligator Snapping Turtles. (PDF)
