# Correction: Different mechanisms of serum complement activation in the plasma of common (*Chelydra serpentina*) and alligator (*Macrochelys temminckii*) snapping turtles

**DOI:** 10.1371/journal.pone.0219772

**Published:** 2019-07-10

**Authors:** Sarah Baker, Ethan Kessler, Lancia Darville, Mark Merchant

The third author’s name is spelled incorrectly. The correct name is: Lancia Darville. The correct citation is: Baker S, Kessler E, Darville L, Merchant M (2019) Different mechanisms of serum complement activation in the plasma of common (*Chelydra serpentina*) and alligator (*Macrochelys temminckii*) snapping turtles. PLoS ONE 14(6): e0217626. https://doi.org/10.1371/journal.pone.0217626.
