# Supplementary material for: Different mechanisms of serum complement activation in the plasma of common (Chelydra serpentina) and alligator (Macrochelys temminckii) snapping turtles
Source: PLoS One. 2019 Jun 6;14(6):e0217626. doi: 10.1371/journal.pone.0217626 (PMC6553747; doi:10.1371/journal.pone.0217626)
Supplement: S1 Fig — (PDF) [file pone.0217626.s001.pdf]

| | | | | | | | | |
|---|---|---|---|---|---|---|---|---|
| | | 0 | 1 | 2 | 3 | 4 | 5 | 6 |
| | common | 0.055 | 0.369 | 0.748 | 0.825 | 0.844 | 0.873 | 0.891 |
| | common | 0.067 | 0.421 | 0.75 | 0.84 | 0.869 | 0.88 | 0.882 |
| | common | 0.057 | 0.379 | 0.716 | 0.833 | 0.849 | 0.865 | 0.877 |
| | common | 0.064 | 0.037 | 0.738 | 0.828 | 0.875 | 0.883 | 0.886 |
| | AST | 0.061 | 0.063 | 0.157 | 0.24 | 0.381 | 0.575 | 0.693 |
| | AST | 0.057 | 0.063 | 0.107 | 0.258 | 0.429 | 0.584 | 0.691 |
| | AST | 0.058 | 0.068 | 0.133 | 0.281 | 0.41 | 0.577 | 0.683 |
| | AST | 0.06 | 0.06 | 0.121 | 0.234 | 0.436 | 0.569 | 0.736 |
| blank | | | | | | | | |
| 0.037 | | | | | | | | |
| | common | 0.018 | 0.332 | 0.711 | 0.788 | 0.807 | 0.836 | 0.854 |
| | common | 0.03 | 0.384 | 0.713 | 0.803 | 0.832 | 0.843 | 0.845 |
| minus blank | common | 0.02 | 0.342 | 0.679 | 0.796 | 0.812 | 0.828 | 0.84 |
| | common | 0.027 | 0 | 0.701 | 0.791 | 0.838 | 0.846 | 0.849 |
| | AST | 0.024 | 0.026 | 0.12 | 0.203 | 0.344 | 0.538 | 0.656 |
| | AST | 0.02 | 0.026 | 0.07 | 0.221 | 0.392 | 0.547 | 0.654 |
| | AST | 0.021 | 0.031 | 0.096 | 0.244 | 0.373 | 0.54 | 0.646 |
| | AST | 0.023 | 0.023 | 0.084 | 0.197 | 0.399 | 0.532 | 0.699 |
| zero | | 0.022875 | | | | | | |
| | | | | | | | | |
| minus zero | common | -0.00488 | 0.309125 | 0.688125 | 0.765125 | 0.784125 | 0.813125 | 0.831125 |
| | common | 0.007125 | 0.361125 | 0.690125 | 0.780125 | 0.809125 | 0.820125 | 0.822125 |
| | common | -0.00288 | 0.319125 | 0.656125 | 0.773125 | 0.789125 | 0.805125 | 0.817125 |
| | common | 0.004125 | -0.02288 | 0.678125 | 0.768125 | 0.815125 | 0.823125 | 0.826125 |
| | AST | 0.001125 | 0.003125 | 0.097125 | 0.180125 | 0.321125 | 0.515125 | 0.633125 |
| | AST | -0.00288 | 0.003125 | 0.047125 | 0.198125 | 0.369125 | 0.524125 | 0.631125 |
| | AST | -0.00188 | 0.008125 | 0.073125 | 0.221125 | 0.350125 | 0.517125 | 0.623125 |
| | AST | 0.000125 | 0.000125 | 0.061125 | 0.174125 | 0.376125 | 0.509125 | 0.676125 |
| | | | | | | | | |
| % max | common | -0.54167 | 34.34722 | 76.45833 | 85.01389 | 87.125 | 90.34722 | 92.34722 |
| | common | 0.791667 | 40.125 | 76.68056 | 86.68056 | 89.90278 | 91.125 | 91.34722 |
| | common | -0.31944 | 35.45833 | 72.90278 | 85.90278 | 87.68056 | 89.45833 | 90.79167 |
| | common | 0.458333 | -2.54167 | 75.34722 | 85.34722 | 90.56944 | 91.45833 | 91.79167 |
| | AST | 0.125 | 0.347222 | 10.79167 | 20.01389 | 35.68056 | 57.23611 | 70.34722 |
| | AST | -0.31944 | 0.347222 | 5.236111 | 22.01389 | 41.01389 | 58.23611 | 70.125 |
| | AST | -0.20833 | 0.902778 | 8.125 | 24.56944 | 38.90278 | 57.45833 | 69.23611 |
| | AST | 0.013889 | 0.013889 | 6.791667 | 19.34722 | 41.79167 | 56.56944 | 75.125 |
| | | | | | | | | |
| | | 0 | 10 | 20 | 30 | 40 | 50 | 60 |
| | common | 0.097222 | 36.64352 | 75.34722 | 85.73611 | 88.81944 | 90.59722 | 91.56944 |
| | | 0 | 3.065808 | 1.730862 | 0.728604 | 1.67375 | 0.890623 | 0.660465 |
| | | | | | | | | |
| | AST | 0 | 0.402778 | 7.736111 | 21.48611 | 39.34722 | 57.375 | 71.20833 |
| | | 0 | 0.368514 | 2.354402 | 2.347181 | 2.732219 | 0.687184 | 2.654874 |

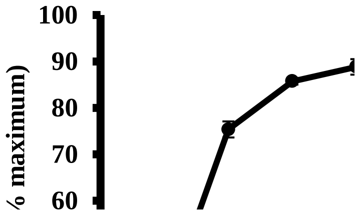

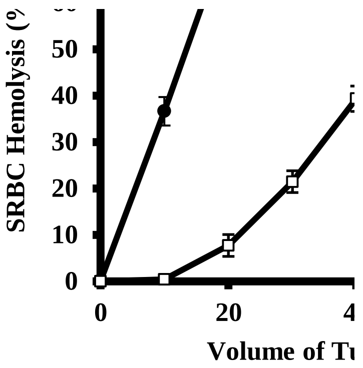
SRBC Hemolysis (%
50
40
30
20
10
0
0
20
4
Volume of Tu

| | | | |
|---|---|---|---|
| 7 | 8 | 9 | 10 |
| 0.893 | 0.912 | 0.921 | 0.934 |
| 0.909 | 0.908 | 0.919 | 0.918 |
| 0.887 | 0.91 | 0.921 | 0.937 |
| 0.9 | 0.904 | 0.92 | 0.93 |
| 0.688 | 0.733 | 0.846 | 0.864 |
| 0.738 | 0.74 | 0.835 | 0.884 |
| 0.738 | 0.77 | 0.799 | 0.867 |
| 0.773 | 0.779 | 0.818 | 0.891 |

| | | | |
|---|---|---|---|
| 0.856 | 0.875 | 0.884 | 0.897 |
| 0.872 | 0.871 | 0.882 | 0.881 |
| 0.85 | 0.873 | 0.884 | 0.9 |
| 0.863 | 0.867 | 0.883 | 0.893 |
| 0.651 | 0.696 | 0.809 | 0.827 |
| 0.701 | 0.703 | 0.798 | 0.847 |
| 0.701 | 0.733 | 0.762 | 0.83 |
| 0.736 | 0.742 | 0.781 | 0.854 |

| | | | |
|---|---|---|---|
| 0.833125 | 0.852125 | 0.861125 | 0.874125 |
| 0.849125 | 0.848125 | 0.859125 | 0.858125 |
| 0.827125 | 0.850125 | 0.861125 | 0.877125 |
| 0.840125 | 0.844125 | 0.860125 | 0.870125 |
| 0.628125 | 0.673125 | 0.786125 | 0.804125 |
| 0.678125 | 0.680125 | 0.775125 | 0.824125 |
| 0.678125 | 0.710125 | 0.739125 | 0.807125 |
| 0.713125 | 0.719125 | 0.758125 | 0.831125 |

| | | | |
|---|---|---|---|
| 92.56944 | 94.68056 | 95.68056 | 97.125 |
| 94.34722 | 94.23611 | 95.45833 | 95.34722 |
| 91.90278 | 94.45833 | 95.68056 | 97.45833 |
| 93.34722 | 93.79167 | 95.56944 | 96.68056 |
| 69.79167 | 74.79167 | 87.34722 | 89.34722 |
| 75.34722 | 75.56944 | 86.125 | 91.56944 |
| 75.34722 | 78.90278 | 82.125 | 89.68056 |
| 79.23611 | 79.90278 | 84.23611 | 92.34722 |

| | | | |
|---|---|---|---|
| 70 | 80 | 90 | 100 |
| 93.04167 | 94.29167 | 95.59722 | 96.65278 |
| 1.05165 | 0.379517 | 0.106381 | 0.926851 |
| | | | |
| 74.93056 | 77.29167 | 84.95833 | 90.73611 |
| 3.885581 | 2.491962 | 2.281614 | 1.452966 |

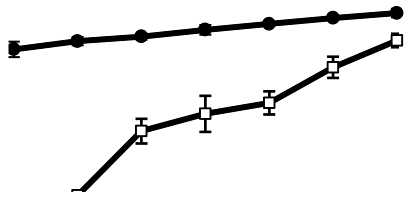

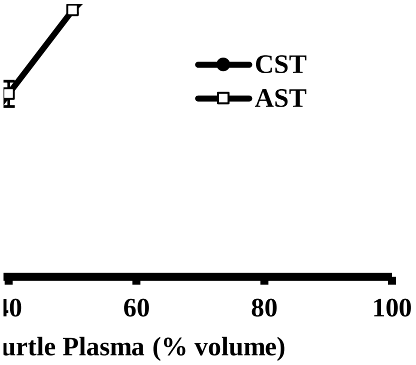
CST
AST
40
60
80
100
urtle Plasma (% volume)
