# Supplementary figures and images for: Different mechanisms of serum complement activation in the plasma of common (Chelydra serpentina) and alligator (Macrochelys temminckii) snapping turtles
Source: PLoS One. 2019 Jun 6;14(6):e0217626. doi: 10.1371/journal.pone.0217626 (PMC6553747; doi:10.1371/journal.pone.0217626)

|                      |        | 0            | 2        | 5        | 10       | 15       | 20       | 30       |
|----------------------|--------|--------------|----------|----------|----------|----------|----------|----------|
|                      | AST    | 0.069        | 0.069    | 0.071    | 0.067    | 0.079    | 0.082    | 0.092    |
|                      |        | 0.068        | 0.068    | 0.064    | 0.068    | 0.076    | 0.08     | 0.092    |
|                      |        | 0.069        | 0.068    | 0.069    | 0.069    | 0.078    | 0.081    | 0.097    |
|                      |        | 0.071        | 0.09     | 0.065    | 0.065    | 0.074    | 0.075    | 0.089    |
|                      | common | 0.084        | 0.088    | 0.133    | 0.304    | 0.483    | 0.588    | 0.666    |
|                      |        | 0.081        | 0.087    | 0.133    | 0.302    | 0.486    | 0.598    | 0.672    |
|                      |        | 0.079        | 0.088    | 0.135    | 0.306    | 0.482    | 0.584    | 0.66     |
|                      |        | 0.08         | 0.087    | 0.136    | 0.309    | 0.492    | 0.598    | 0.676    |
| minus blank<br>0.037 | AST    | 0.032        | 0.032    | 0.034    | 0.03     | 0.042    | 0.045    | 0.055    |
|                      |        | 0.031        | 0.031    | 0.027    | 0.031    | 0.039    | 0.043    | 0.055    |
|                      |        | 0.032        | 0.031    | 0.032    | 0.032    | 0.041    | 0.044    | 0.06     |
|                      |        | 0.034        | 0.053    | 0.028    | 0.028    | 0.037    | 0.038    | 0.052    |
|                      | common | 0.03225      |          |          |          |          |          |          |
|                      |        | 0.047        | 0.051    | 0.096    | 0.267    | 0.446    | 0.551    | 0.629    |
|                      |        | 0.044        | 0.05     | 0.096    | 0.265    | 0.449    | 0.561    | 0.635    |
|                      |        | 0.042        | 0.051    | 0.098    | 0.269    | 0.445    | 0.547    | 0.623    |
| minus zero           | AST    | 0.043        | 0.05     | 0.099    | 0.272    | 0.455    | 0.561    | 0.639    |
|                      |        | 0.044        |          |          |          |          |          |          |
|                      |        | -0.00025     | -0.00025 | 0.00175  | -0.00225 | 0.00975  | 0.01275  | 0.02275  |
|                      |        | -0.00125     | -0.00125 | -0.00525 | -0.00125 | 0.00675  | 0.01075  | 0.02275  |
|                      | common | -0.00025     | -0.00125 | -0.00025 | -0.00025 | 0.00875  | 0.01175  | 0.02775  |
|                      |        | 0.00175      | 0.02075  | -0.00425 | -0.00425 | 0.00475  | 0.00575  | 0.01975  |
|                      |        | 0.003        | 0.007    | 0.052    | 0.223    | 0.402    | 0.507    | 0.585    |
|                      |        | 0            | 0.006    | 0.052    | 0.221    | 0.405    | 0.517    | 0.591    |
| % max                | AST    | -0.002       | 0.007    | 0.054    | 0.225    | 0.401    | 0.503    | 0.579    |
|                      |        | -0.001       | 0.006    | 0.055    | 0.228    | 0.411    | 0.517    | 0.595    |
|                      |        | 1.220275     | 1.595745 | 2.847309 |          |          |          |          |
|                      |        | 0.844806     | 1.345432 | 2.847309 |          |          |          |          |
|                      | common | 1.095119     | 1.470588 | 3.473091 |          |          |          |          |
|                      |        | 0.594493     | 0.71965  | 2.47184  |          |          |          |          |
|                      |        | 0            | 0        | 0        | 0        | 0.938673 | 1.282854 | 2.909887 |
|                      |        | 0            | 0        | 0        | 0        | 0.277516 | 0.389127 | 0.415097 |
|                      | AST    | 0.375469337  | 0.876095 | 6.508135 | 27.90989 | 50.31289 | 63.45432 | 73.21652 |
|                      |        | 0            | 0.750939 | 6.508135 | 27.65957 | 50.68836 | 64.70588 | 73.96746 |
|                      |        | -0.250312891 | 0.876095 | 6.758448 | 28.1602  | 50.18773 | 62.95369 | 72.46558 |
|                      |        | -0.125156446 | 0.750939 | 6.883605 | 28.53567 | 51.4393  | 64.70588 | 74.46809 |
|                      | common | 0            | 0.813517 | 6.664581 | 28.06633 | 50.65707 | 63.95494 | 73.52941 |
|                      |        | 0            | 0.072259 | 0.187735 | 0.373727 | 0.563204 | 0.89087  | 0.876095 |
|                      |        |              |          |          |          |          |          |          |
|                      |        |              |          |          |          |          |          |          |

100  
90  
80  
70  
is (%)

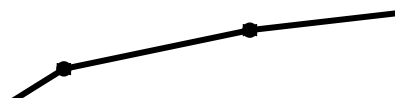

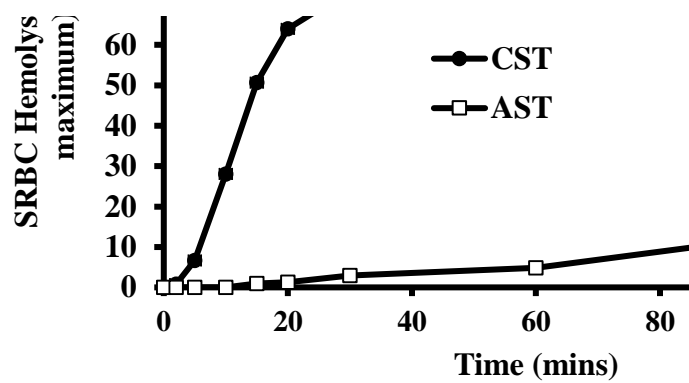

| 60    | 90    | 120   |
|-------|-------|-------|
| 0.107 | 0.172 | 0.172 |
| 0.107 | 0.154 | 0.175 |
| 0.11  | 0.149 | 0.176 |
| 0.108 | 0.149 | 0.177 |
| 0.745 | 0.783 | 0.836 |
| 0.756 | 0.795 | 0.8   |
| 0.736 | 0.786 | 0.811 |
| 0.745 |       |       |

|       |       |       |
|-------|-------|-------|
| 0.07  | 0.135 | 0.135 |
| 0.07  | 0.117 | 0.138 |
| 0.073 | 0.112 | 0.139 |
| 0.071 | 0.112 | 0.14  |

|       |       |       |
|-------|-------|-------|
| 0.708 | 0.746 | 0.799 |
| 0.719 | 0.758 | 0.763 |
| 0.699 | 0.749 | 0.774 |
| 0.708 |       |       |

|         |         |         |
|---------|---------|---------|
| 0.03775 | 0.10275 | 0.10275 |
| 0.03775 | 0.08475 | 0.10575 |
| 0.04075 | 0.07975 | 0.10675 |
| 0.03875 | 0.07975 | 0.10775 |

|       |       |       |
|-------|-------|-------|
| 0.664 | 0.702 | 0.755 |
| 0.675 | 0.714 | 0.719 |
| 0.655 | 0.705 | 0.73  |
| 0.664 |       |       |

|          |          |          |
|----------|----------|----------|
| 4.724656 | 12.85982 | 12.85982 |
| 4.724656 | 10.60701 | 13.23529 |
| 5.100125 | 9.981227 | 13.36045 |
| 4.849812 | 9.981227 | 13.48561 |
| 4.849812 | 10.85732 | 13.23529 |
| 0.176998 | 1.367206 | 0.270369 |

|          |          |          |
|----------|----------|----------|
| 83.10388 | 87.85982 | 94.49312 |
| 84.4806  | 89.3617  | 89.98748 |
| 81.97747 | 88.23529 | 91.36421 |
| 83.10388 |          |          |
| 83.16646 | 88.48561 | 91.94827 |
| 1.02445  | 0.781602 | 2.308902 |

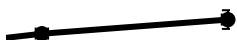

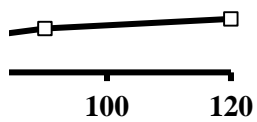

Supplement: S2 Fig — (PDF) [file pone.0217626.s002.pdf]
