# Supplementary material for: Different mechanisms of serum complement activation in the plasma of common (Chelydra serpentina) and alligator (Macrochelys temminckii) snapping turtles
Source: PLoS One. 2019 Jun 6;14(6):e0217626. doi: 10.1371/journal.pone.0217626 (PMC6553747; doi:10.1371/journal.pone.0217626)

		5	10	15	20	25	30
plasma too low	AST	0.069	0.074	0.076	0.087	0.107	0.137
	AST	0.071	0.072	0.072	0.088	0.108	0.135
	AST	0.075	0.074	0.077	0.085	0.109	0.147
	AST	0.071	0.076	0.076	0.081	0.109	0.126
	common	0.064	0.075	0.092	0.18	0.263	0.218
	common	0.061	0.072	0.089	0.181	0.241	0.22
	common	0.065	0.073	0.1	0.18	0.235	0.218
	common	0.063	0.072	0.096	0.18	0.235	0.208
		5	10	15	20	25	30
plasma JUST RIGHT	AST	0.167	0.254	0.475	0.651	0.85	0.913
	AST	0.129	0.267	0.46	0.653	0.859	0.907
	AST	0.146	0.258	0.502	0.637	0.854	0.863
	AST	0.131	0.274	0.501	0.611	0.863	0.858
	common	0.179	0.445	0.768	0.82	0.845	0.873
	common	0.19	0.407	1.122	0.814	0.872	0.853
	common	0.163	0.432	0.801	0.778	0.846	0.836
	common	0.197	0.407	0.829	0.785	0.871	0.865
minus blank	AST	0.131	0.218	0.439	0.615	0.814	0.877
0.036	AST	0.093	0.231	0.424	0.617	0.823	0.871
	AST	0.11	0.222	0.466	0.601	0.818	0.827
	AST	0.095	0.238	0.465	0.575	0.827	0.822
	common	0.143	0.409	0.732	0.784	0.809	0.837
	common	0.154	0.371	1.086	0.778	0.836	0.817
	common	0.127	0.396	0.765	0.742	0.81	0.8
	common	0.161	0.371	0.793	0.749	0.835	0.829
% max	AST	16.07362	26.74847	53.86503	75.46012	99.8773	107.6074
	AST	11.41104	28.34356	52.02454	75.70552	100.9816	106.8712
	AST	13.49693	27.23926	57.17791	73.74233	100.3681	101.4724
	AST	11.65644	29.20245	57.05521	70.55215	101.4724	100.8589
		13.15951	27.88344	55.03067	73.86503	100.6748	104.2025
		2.154239	1.103726	2.523554	2.375007	0.697698	3.528363
	common	17.54601	50.18405	89.81595	96.19632	99.2638	102.6994
	common	18.89571	45.52147	133.2515	95.46012	102.5767	100.2454
	common	15.58282	48.58896	93.86503	91.04294	99.3865	98.15951
	common	19.7546	45.52147	97.30061	91.90184	102.454	101.7178
		17.94479	47.45399	93.66053	93.65031	100.9202	100.7055
		1.818202	2.324552	3.746519	2.556892	1.843215	1.974342

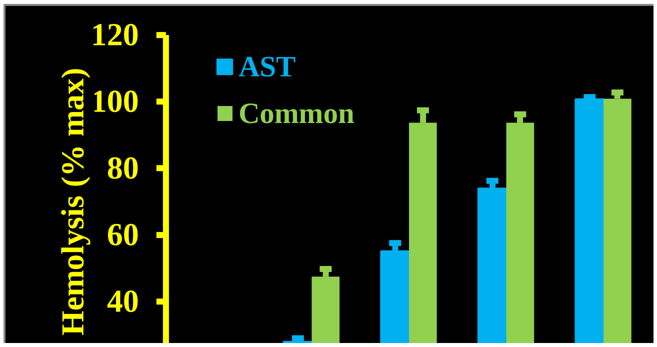

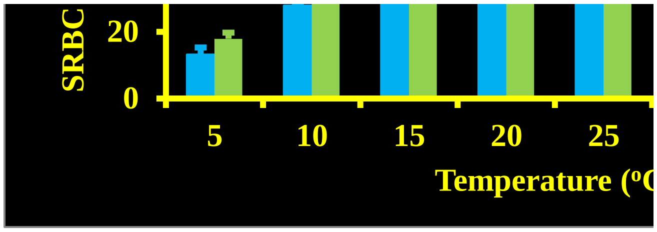
SRBC
20
0
5
10
15
20
25
Temperature (ºC

35	40
0.161	0.086
0.165	0.088
0.194	0.086
0.172	0.271
0.213	0.146
0.207	0.15
0.216	0.15
0.215	0.154
35	40
0.834	0.655
0.848	0.659
0.864	0.615
0.851	0.616
0.731	0.772
0.781	0.781
0.759	0.753
0.749	0.749
0.798	0.619
0.812	0.623
0.828	0.579
0.815	0.58
0.695	0.736
0.745	0.745
0.723	0.717
0.713	0.713
97.91411	75.95092
99.6319	76.44172
101.5951	71.04294
100	71.16564
99.78528	73.65031
1.510665	2.947128
85.27607	90.30675
91.41104	91.41104
88.71166	87.97546
87.48466	87.48466
88.22086	89.29448
2.558118	1.87259

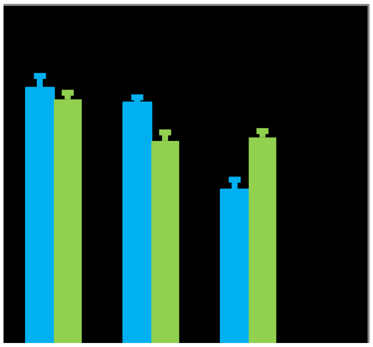

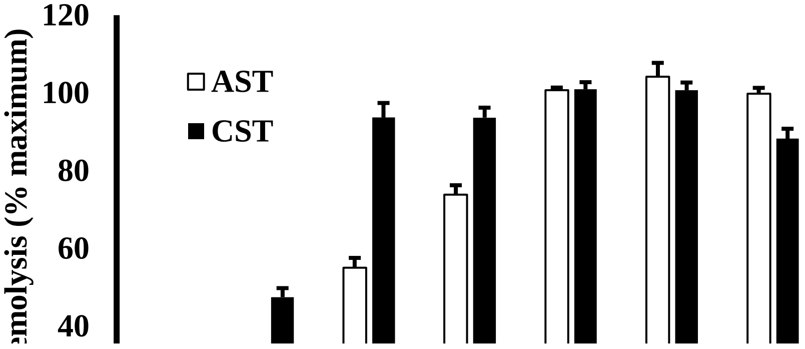

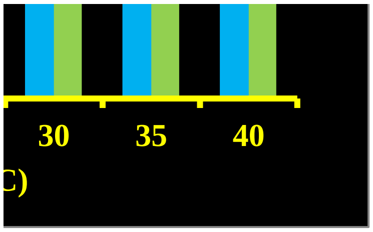

30
35
40
C)

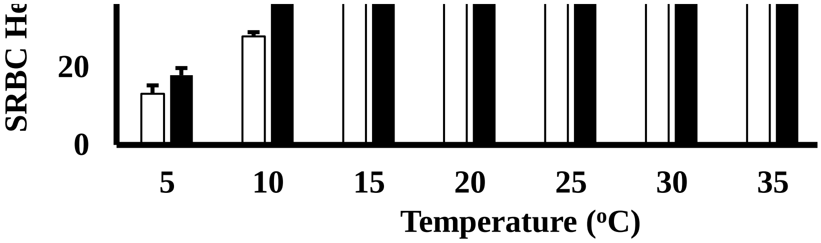

SRBC He
20
0
5
10
15
20
25
30
35
Temperature (oC)

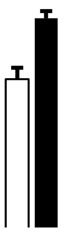

Supplement: S3 Fig — (PDF) [file pone.0217626.s003.pdf]
